# Risks of anxiety disorders, depressive disorders, and sleep disorders in patients with dengue fever: A nationwide, population-based cohort study

**DOI:** 10.1371/journal.pntd.0012239

**Published:** 2024-07-03

**Authors:** Hsin-I Shih, Yu-Ping Wang, Chia-Yu Chi, Yu-Wen Chien

**Affiliations:** 1 Department of Emergency Medicine, National Cheng Kung University Hospital, College of Medicine, National Cheng Kung University, Tainan, Taiwan; 2 School of Medicine, College of Medicine, National Cheng Kung University, Tainan, Taiwan; 3 Department of Public Health, College of Medicine, National Cheng Kung University, Tainan, Taiwan; 4 National Mosquito-Borne Diseases Control Research Center, National Health Research Institutes, Miaoli County, Taiwan; 5 Department of Microbiology & Immunology, College of Medicine, National Cheng Kung University, Tainan, Taiwan; 6 Department of Occupational and Environmental Medicine, National Cheng Kung University Hospital, College of Medicine, National Cheng Kung University, Tainan, Taiwan; University of Rhode Island, UNITED STATES

## Abstract

**Background:**

Dengue virus (DENV) infection, a common mosquito-borne disease, has been linked to several mental disorders like depression and anxiety. However, the temporal risk of these disorders after DENV infection is not well studied.

**Methods:**

This population-based cohort study encompassed 45,334 recently lab-confirmed dengue patients in Taiwan spanning 2002 to 2015, matched at a 1:5 ratio with non-dengue individuals based on age, gender, and residence (n = 226,670). Employing subdistribution hazard regression analysis, we assessed the immediate (<3 months), intermediate (3–12 months), and prolonged (>12 months) risks of anxiety disorders, depressive disorders, and sleep disorders post DENV infection. Corrections for multiple comparisons were carried out using the Benjamini-Hochberg procedure.

**Results:**

A significant increase in depressive disorder risk across all timeframes post-infection was observed (<3 months [aSHR 1.90, 95% CI 1.20–2.99], 3–12 months [aSHR 1.68, 95% CI 1.32–2.14], and >12 months [aSHR 1.14, 95% CI 1.03–1.25]). Sleep disorder risk was higher only during 3–12 months (aSHR 1.55, 95% CI 1.18–2.04). No elevated anxiety disorder risk was found. Subgroup analysis of hospitalized dengue patients showed increased risk of anxiety disorders within 3 months (aSHR 2.14, 95% CI 1.19–3.85) and persistent risk of depressive disorders across all periods. Hospitalized dengue patients also had elevated sleep disorder risk within the first year.

**Conclusion:**

Dengue patients exhibited significantly elevated risks of depressive disorders in both the short and long term. However, dengue’s impact on sleep disorders and anxiety seems to be short-lived. Further research is essential to elucidate the underlying mechanisms.

## Author summary

Dengue fever is a mosquito-borne tropical disease caused by the dengue virus. Several DENV-induced psychiatric disorders such as anxiety and depression have been identified. Most of the previous studies conducted in the acute stage. Whether deengue infection has lonng-term effects on mental stress remains obscure. Our study suggested that a significant increase in depressive disorder risk across all timeframes post-infection: <3 months, 3–12 months, and >12 months. Sleep disorder risk was higher only during 3–12 months. However, no elevated anxiety disorder risk was found.

## Introduction

Dengue virus, a member of the *Flavivirus* genus within the Flaviviridae family, is an arthropod-borne virus that comprises four distinct serotypes (DENV-1, DENV-2, DENV-3, and DENV-4) [1]. The World Health Organization (WHO) considers dengue as a major global public health challenge in tropical and subtropical regions. Individuals infected with DENV exhibit a broad spectrum of clinical manifestations, which can range from being asymptomatic to developing dengue fever (DF) or severe dengue (SD), formerly recognized as dengue hemorrhagic fever (DHF) and dengue shock syndrome (DSS) [2]. Severe dengue typically develops after the febrile phase and is characterized by gastrointestinal bleeding, plasma leakage, shock, respiratory distress, or multi-organ failure. The critical phase requires management with judicious intravenous fluid replacement; some of the patients may necessitate complex intensive supportive care. Following the critical phase, patients transition into the convalescence phase [3], which in adults can last 2–4 weeks; some patients may accompanied with fatigue symptoms [3]. Following the convalescent phase, several follow-up studies suggest that certain individuals recovering from dengue may experience persistent symptoms, including fever, headache, skin rash, and chronic fatigue, which can persist for up to two months or even extend to two years post-infection [4–6]. Therefore, DENV infection may result in long-term health consequences for some individuals.

Several DENV-induced psychiatric disorders have been reported previously [7, 8]. Elevated levels of inflammatory cytokines in depression patients were also observed in dengue patients [7,9–14]. Some studies have suggested a high prevalence (up to 60%) of depression and anxiety symptoms [15,16]. The severity of fever, headache, myalgias and arthralgias, and retro/periorbital pain were all positively correlated with both anxiety and depression with the strongest correlation between severity of headache and scores for both anxiety and depression [15]. Moreover, dengue patients have been observed to develop phobic disorders and post-traumatic stress disorder [17,18]. However, these studies have predominantly been conducted in the acute clinical setting within hospitals, where physical illness and hospitalization stressors may have also contributed to these acute psychological manifestations. The long-term effects of dengue on mental stress remain obscure.

The primary objective of this study was to investigate the short-term, mid-term, and long-term risks associated with three mental disorders—specifically, anxiety disorders, depressive disorders, and sleep disorders—following a DENV infection. Taiwan, situated in East Asia and home to over 23 million people, has traditionally seen relatively minor dengue outbreaks, usually recording only a few hundred to a thousand cases annually before 2014. These outbreaks have been predominantly located in the southern areas, including Tainan, Kaohsiung, and Pingtung. Notably, the years 2014 and 2015 marked a significant deviation, with outbreaks escalating to more than 15,000 and 43,000 cases, respectively. Types 1 and 2 are the most prevalent dengue serotypes in Taiwan [19]. Despite the regularity of these outbreaks, dengue has yet to be classified as endemic in Taiwan, maintaining a comparatively low overall seroprevalence [20–22]. This is distinctly different from hyperendemic regions, where the vast majority have undergone multiple DENV infections, rendering the selection of uninfected control individuals for study purposes challenging. The availability of extensive national health databases, coupled with the relatively low dengue incidence in Taiwan, offers a distinct and invaluable framework for exploring the proposed hypothesis.

## Materials and methods

### Ethics statement

This study was commenced after obtaining approval from the Institutional Review Board of National Cheng Kung University Hospital (B-ER-106-184). This study was performed in accordance with the Helsinki Declaration of 1964 and its later amendments. Because the data were deidentified and analyzed for research purposes, the need for informed consent was waived.

### Data source and study population

Taiwan’s National Health Insurance (NHI) Program, established in 1995, provides comprehensive medical coverage to all citizens and eligible foreign residents, with a remarkable coverage rate exceeding 99%. The sustained and extensive accumulation of NHI-claimed data has played a crucial role in the establishment of the National Health Insurance Research Database (NHIRD), which presently serves as a fundamental platform for big data analysis in Taiwan’s healthcare-related fields [23]. The Health and Welfare Data Science Center, operating under the Ministry of Health and Welfare (HWDC, MOHW), maintains a secure data repository encompassing the NHIRD along with various other health and welfare-related databases. This repository is exclusively utilized for academic research projects that have been approved by research ethics committees. To ensure confidentiality and security, rigorous data protection policies are in place, including encrypting individual identification numbers, conducting supervised on-site analyses, and performing meticulous manual reviews of analysis results [23].

Newly laboratory-confirmed cases of dengue fever between 2002 and 2015 were identified from the Notifiable Disease Dataset of Confirmed Cases (NDDCC), provided by the Taiwan Centers for Disease Control. During the study period, a case was considered laboratory-confirmed if any one of the following criteria was met: 1) isolation of DENV, 2) positive results from real-time reverse transcription-polymerase chain reaction tests, 3) detection of high-titer dengue-specific IgM or IgG antibodies in single serum samples (up until 2009), 4) a four-fold increase in IgG titer between paired acute and convalescent-phase serum samples, and 5) positive nonstructural protein 1 (NS1) test results [24,25]. For each selected case, the date of symptom onset was designated as the index date. Cases were excluded based on: 1) missing or invalid national identification number; 2) any prior laboratory-confirmed diagnosis between 1998 and the index date in the dataset; 3) uncertain or incomplete personal information, such as gender, birth year, or residential area; 4) death within 30 days post the index date; and 5) any previous diagnosis of anxiety disorders, depressive disorders, and sleep disorders before the index date (S1 Table), irrespective of the diagnosing physician’s specialty or the diagnosis frequency or setting. For each dengue case, five nondengue controls were randomly selected, matched based on age, sex, residential area, and the index year and month. The same exclusion criteria were applied to the nondengue population as with the dengue cases.

### Study outcome and follow-up

This study aimed to investigate whether dengue patients had an increased risk of the following three mental diseases: anxiety disorders, depressive disorders, and sleep disorders. Outcomes were identified by at least one hospital admission or a minimum of three outpatient clinic visits, with relevant diagnostic codes (ICD-9-CM or ICD-10-CM) determined by board-certified psychiatrists (S1 Table). The follow-up for all dengue cases and nondengue subjects in the study cohort was initiated from the index dates until the first occurrence of any outcome, death, or the end of 2018, whichever occurred first.

### Covariates

The sociodemographic variables considered in the analysis included sex, age, administrative region of residence, monthly income, and the degree of urbanization, with 1 indicating the most urbanized and 4 representing the least urbanized as previously described [26,27]. In addition, the Charlson Comorbidity Index (CCI) with the modified score weighting scheme was calculated as a proxy measure of overall health status [28]. The CCI is a validated method for classifying comorbid conditions which might alter the prognosis of patients in longitudinal studies. It assigns weights to a range of comorbidities, with higher scores indicating a greater burden of comorbid conditions and a higher risk of mortality [29].

### Statistical analysis

We used the standardized mean difference (SMD) as the summary measure to quantify the difference between the groups with and without dengue; a difference was considered meaningful only when the SMD value surpassed 0.1 [30]. The incidence rates of the three mental diseases were calculated by dividing the number of events that occurred after the index dates by the total person-time in years that was accumulated from all subjects who were followed up. We utilized the Kaplan-Meier approach to plot the cumulative occurrence of mental illness in both the dengue and non-dengue groups. The association between DENV infection and each mental disorder was assessed by utilizing the subdistribution hazard ratio (SHR) along with the corresponding 95% confidence intervals (CIs), as derived from the Fine-Gray subdistribution hazard model, adjusting for the aforementioned potential confounders and competing risk of death. Interaction terms were introduced to investigate whether the relationship between dengue infection and mental disorders varied by sex and age. We further performed stratified analyses by follow-up time to compare the short-term (< 3 months), medium-term (3 ~ 12 months), and long-term (>12 months) risk of mental diseases between people with and without dengue. Additionally, subgroup analyses focusing on hospitalized cases were performed because the severity of dengue might affect the risk of study outcomes. To account for multiple comparison, the Benjamini-Hochberg (BH) procedure was employed to control false discovery rate at level 0.05 [31]. SAS 9.4 (SAS Institute, Cary, NC) was used for data processing and analyses in this study.

## Results

**Fig 1:**
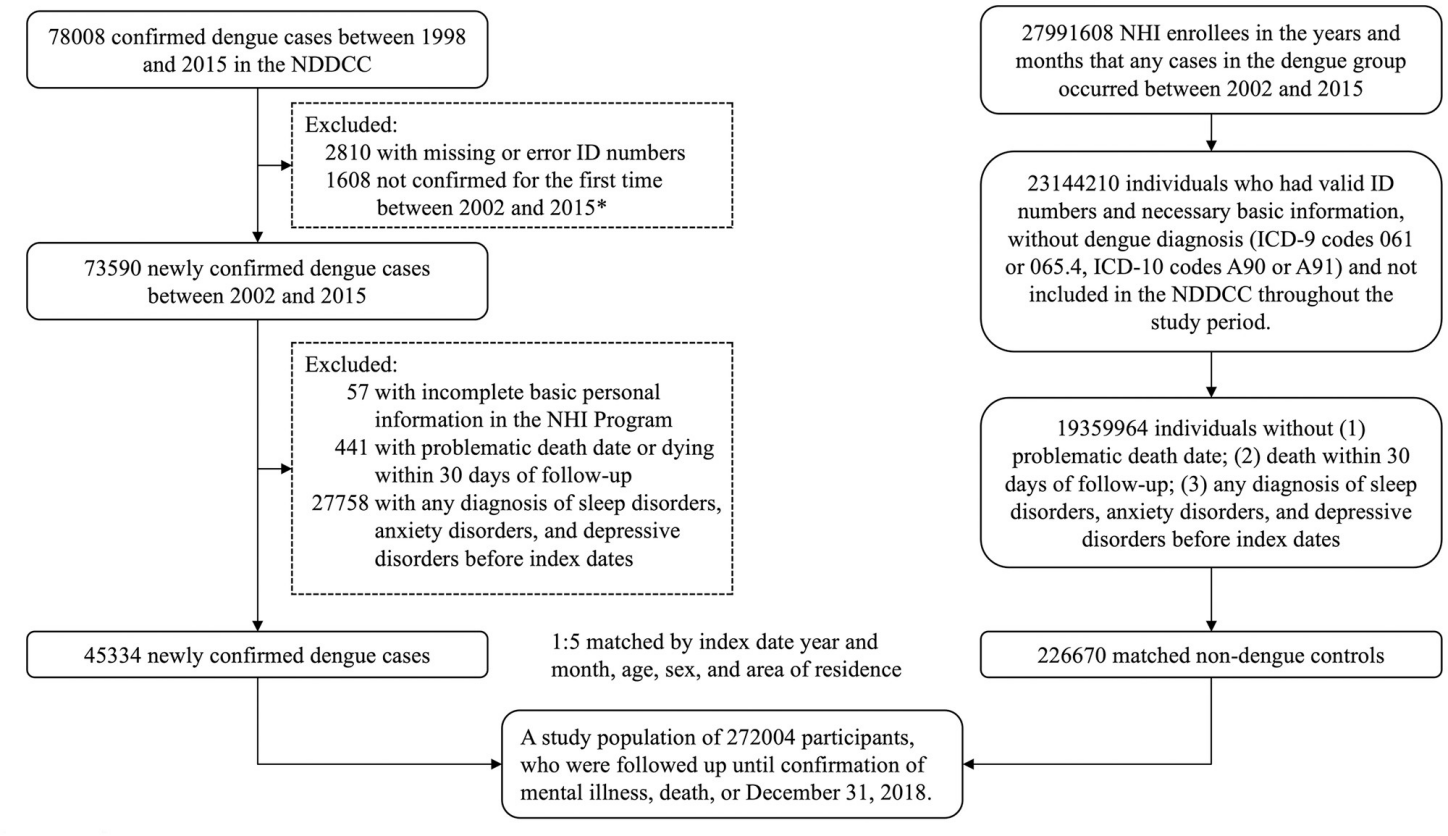
The selection process for the study cohort. *These 1608 cases include (1) cases initially diagnosed from 1998 to 2001, and (2) cases from 2002 to 2015 who had prior laboratory-confirmed dengue infections.

A total of 45,334 eligible dengue cases between 2002 and 2015 and five times of nondengue individuals (226,670) were included in this study (Fig 1). The mean age with standard deviation was 39.2 ± 20.1 years in both groups. The median follow-up time for the dengue and non-dengue groups was the same (3.33 years, IQR 3.21–4.31). Table 1 shows the baseline characteristics for both groups. Dengue patients tended to live in more urbanized areas compared to those without dengue. No significant differences were detected between the two groups for other baseline characteristics, such as monthly income and CCI score.

**Table 1:** Demographic and clinical characteristics in dengue and non-dengue groups.

	Dengue cohort (n = 45,334)	Non-dengue cohort (n = 226,670)	SMD
Sex			
Female	19,936 (44.0)	99,680 (44.0)	-
Male	25,398 (56.0)	126,990 (56.0)	-
Age (years)	39.2 ± 20.1	39.2 ± 20.1	-
0–17	7,544 (16.6)	37,720 (16.6)	-
18–35	13,204 (29.1)	66,020 (29.1)	-
36–50	9,901 (21.8)	49,505 (21.8)	-
51–64	9,280 (20.5)	46,400 (20.5)	-
≥ 65	5,405 (11.9)	27,025 (11.9)	-
Area of residence			
Tainan	15,877 (35.0)	79,385 (35.0)	-
Kaohsiung	26,794 (59.1)	133,970 (59.1)	-
Pingtung	1,143 (2.5)	5,715 (2.5)	-
Others	1,520 (3.4)	7,600 (3.4)	-
Urbanization			
1	16,001 (35.3)	53,387 (23.6)	0.269
2	18,341 (40.4)	73,328 (32.4)	0.170
3	9,188 (20.3)	62,657 (27.6)	0.167
4–7	1,831 (4.0)	37,298 (16.5)	0.341
Income			
Not-employed	7,813 (17.2)	36,714 (16.2)	0.028
Low	18,870 (41.6)	99,870 (44.1)	0.049
High	18,651 (41.1)	90,086 (39.7)	0.029
CCI			
0	29,382 (64.8)	155,013 (68.4)	0.077
1	9,019 (19.9)	41,104 (18.1)	0.045
≥ 2	6,933 (15.3)	30,553 (13.5)	0.053

Values are presented as number of subjects (%) or mean ± standard deviation unless otherwise noted.

Abbreviation: SMD = standardized mean difference, CCI = Charlson Comorbidity Index.

**Fig 2:**
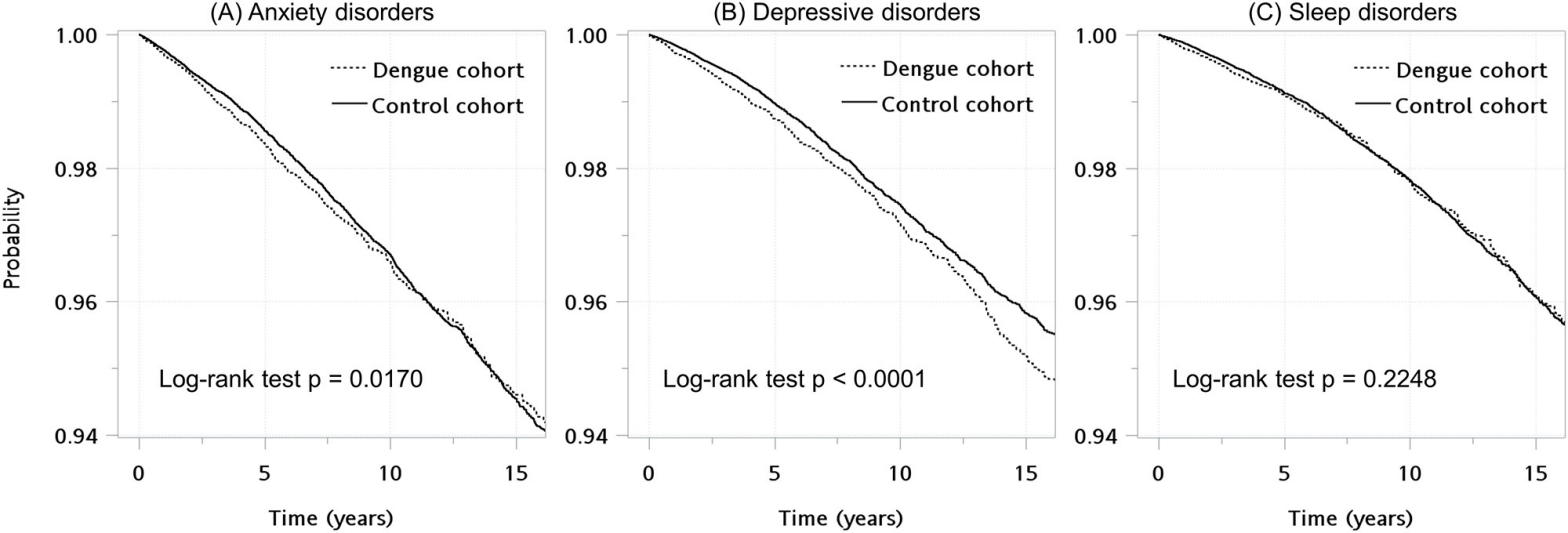
Survival curves of developing (A) anxiety disorders, (B) depressive disorders, and (C) sleep disorders among patients with dengue and control group.

Kaplan-Meier curves indicated a significantly elevated risk of developing anxiety and depressive disorders in dengue patients compared to nondengue subjects; however, there was no such evidence for sleep disorders (Fig 2). The incidence rates of depressive disorders were 273.93 and 223.81 per 100,000 person-years for the dengue and nondengue groups, respectively (Table 2). The Benjamini-Hochberg procedure was used to adjust for the number of tests in Table 2 and Table 3 (n = 24). The Fine-Gray subdistribution hazard model showed a significant association between DENV infection and an increased risk of depressive disorders (adjusted subdistribution hazard ratio, aSHR, 1.21, 95% CI 1.11–1.33, BH-adjusted P < 0.001, Table 2) after adjusting for age, sex, area of residence, urbanization level, income, and CCI score. There was no association found between dengue and sleep disorders or anxiety disorders. No interaction terms with sex and age were statistically significant, therefore, subgroup analyses for sex and age were not carried out.

**Table 2:** Comparison of incidence and hazard ratio of mental illness between dengue and non-dengue cohorts, overall and stratified by follow-up period.

Mental illness	Dengue cohort		Non-dengue cohort			BH-adjusted p-value
	No. of events	IR^a^	No. of events	IR^a^	aSHR^b^ (95% CI)^c^	
Anxiety disorders	801	340.80	3,624	310.18	1.08 (1.00–1.16)	0.101
< 3 months	35	310.11	117	207.29	1.53 (1.03–2.27)	0.063
3~12 months	103	304.24	413	244.00	1.19 (0.96–1.48)	0.183
> 12 months	663	349.15	3,094	328.22	1.05 (0.96–1.14)	0.380
Depressive disorders	645	273.93	2,622	223.81	1.21 (1.11–1.33)	< 0.001
< 3 months	27	239.22	76	134.64	1.90 (1.20–2.99)	0.020
3~12 months	91	268.71	266	157.07	1.68 (1.32–2.14)	< 0.001
> 12 months	527	276.92	2,280	241.08	1.14 (1.03–1.25)	0.022
Sleep disorders	506	214.02	2,366	201.34	1.04 (0.95–1.15)	0.445
< 3 months	22	194.90	65	115.15	1.70 (1.02–2.81)	0.069
3~12 months	69	203.69	210	123.99	1.55 (1.18–2.04)	0.008
> 12 months	415	216.98	2,091	220.26	0.97 (0.87–1.08)	0.629

Abbreviation: PY = person-years, cSHR = crude subdistribution hazard ratio, CI = confidence interval, aSHR = adjusted subdistribution hazard ratio, BH = Benjamini–Hochberg procedure

^a^Incidence rate, per 100,000 person-years.

^b^Adjusted for sex, age, area of residence, urbanization, income, and CCI.

^c^ 95% CIs were not adjusted for multiple comparisons and thus cannot be directly used for hypothesis testing or inference.

**Table 3:** Comparison of incidence and hazard ratio of mental illness between hospitalized dengue cases and non-dengue cohorts, overall and stratified by follow-up period.

Mental illness	Dengue cohort		Non-dengue cohort			
	No. of events	IR^a^	No. of events	IR^a^	aSHR^b^ (95% CI)^c^	BH-adjusted p-value
Anxiety disorders	362	368.46	1,605	329.01	1.08 (0.97–1.22)	0.234
< 3 months	16	413.71	37	191.24	2.14 (1.19–3.85)	0.027
3~12 months	37	319.76	132	227.84	1.31 (0.91–1.91)	0.212
> 12 months	309	373.16	1,436	349.78	1.04 (0.91–1.18)	0.525
Depressive disorders	313	318.16	1,217	248.64	1.26 (1.11–1.43)	0.003
< 3 months	12	310.29	24	124.04	2.62 (1.30–5.32)	0.022
3~12 months	36	311.02	91	156.99	1.90 (1.28–2.82)	0.008
> 12 months	265	319.52	1,102	267.37	1.17 (1.02–1.34)	0.047
Sleep disorders	225	227.19	1,007	204.75	1.07 (0.92–1.24)	0.445
< 3 months	11	284.41	17	87.75	3.00 (1.40–6.42)	0.019
3~12 months	26	224.54	70	120.75	1.69 (1.08–2.64)	0.047
> 12 months	188	224.91	920	221.96	0.98 (0.84–1.15)	0.817

Abbreviation: PY = person-years, cSHR = crude subdistribution hazard ratio, CI = confidence interval, aSHR = adjusted subdistribution hazard ratio, BH = Benjamini–Hochberg procedure

^a^Incidence rate, per 100,000 person-years.

^b^Adjusted for sex, age, area of residence, urbanization, income, and CCI.

^c^ 95% CIs were not adjusted for multiple comparisons and thus cannot be directly used for hypothesis testing or inference.

Additionally, upon stratifying the follow-up duration, we observed that the risk of depressive disorders significantly escalated across all intervals after DENV infection (< 3 months, aSHR 1.90, 95% CI 1.20–2.99, BH-adjusted P = 0.020; 3~12 months, aSHR 1.68, 95% CI 1.32–2.14, BH-adjusted P < 0.001; >12 months, aSHR 1.14, 95% CI 1.03–1.25, BH-adjusted P = 0.022, Table 2). However, an increased risk of sleep disorders was associated with dengue only within 3 to 12 months following DENV infection (aSHR 1.55, 95% CI 1.18–2.04, BH-adjusted P = 0.008). No elevated risk of anxiety disorders was identified at any time period among dengue patients.

Among all the dengue cases, 15,542 (34.3%) patients were admitted to the hospital within 14 days of symptom onset. Subgroup analyses of hospitalized dengue patients revealed an increased risk for anxiety disorders only within 3 months after DENV infection (aSHR 2.14, 95% CI 1.19–3.85, BH-adjusted P = 0.027, Table 3). Hospitalized dengue patients had a higher risk of depressive disorders in all time periods after DENV infection. The risk of sleep disorders significantly increased within one year after the onset of dengue (< 3 months, aSHR 3.00, 95% CI 1.40–6.42, BH-adjusted P = 0.019; 3~12 months, aSHR 1.69, 95% CI 1.08–2.64, BH-adjusted P = 0.047, Table 3). However, this significance disappeared after subjects entered into the long-term follow-up beyond one year.

## Discussion

The most significant finding of our study is the heightened risk of depressive disorders observed in patients after dengue infection, compared to those without dengue. This risk was consistently elevated across different follow-up durations (< 3 months, 3–12 months, and >12 months) post-DENV infection, indicating the potential for dengue infection to trigger some mental illnesses, particularly depression. Moreover, there was a mild elevation in the risk of sleep disorders within three to twelve months after DENV infection, while no heightened risk of anxiety disorders was reported during the study period. Among those hospitalized due to dengue, the risk of the three mental illnesses was more pronounced, particularly within the first three months post-infection. These findings highlight the potential impact of dengue infection on mental health and underscore the importance of further investigation into the mechanisms underlying these associations.

DENV-induced encephalopathy, occurring during the acute phase of the disease, may present as decreased sensitivity, cognitive dysfunction, seizures, and personality disorders, including sudden mania, depression, anxiety, psychosis, and agoraphobia [32]. Proposed mechanisms suggested that brain inflammation can alter serotonin neurotransmitter levels, potentially leading to the development of mood disorders [33]. The neurotoxic inflammation induced by cytokines has been found to decrease tryptophan levels, the amino acid precursor for serotonin, which positively correlates with the severity of depressive symptoms in patients [33,34]. Elevated levels of other inflammatory cytokines such as Interleukin-1 beta (IL-1 β), Interleukin-2 (IL-2), and Interleukin-6 (IL-6) have also been observed in patients with major depressive disorder compared to healthy controls [7,9–14]. Therefore, the presence of these cytokines, absent among controls, may partially explain the higher incidence and severity of depressive symptoms in patients during DENV infection. Reports from Sri Lanka and India have highlighted dengue patients had higher depressive scores, anxiety scores, stress scores than controls [15,16] and about 15% prevalence of depression in the dengue group during a 6 to 24-month follow-up after dengue infection; the anxiety and depressive symptoms correlated with the dengue infection severity [16]. In addition, long-term follow-up studies indicate that certain individuals may experience enduring symptoms, including headache, skin rash, and chronic fatigue, well beyond the acute phase of dengue infection [4–6]. Importantly, these prolonged symptoms have the potential to contribute to the development of long-term depressive conditions post-infection. Furthermore, cerebral involvement and the potential for psychiatric manifestations of dengue in association with clinical and subclinical encephalitis have been indicated [35]. Parallels can be drawn with the West Nile virus (WNV)—another member of the Flavivirus family. Post-infection depression following WNV has been documented in the U.S., with some patients reporting depressive symptoms even a year after contracting the virus [34,36,37]. Given that dengue also falls under the Flavivirus category, it’s plausible to surmise that patients infected with dengue might develop psychiatric disorders post-infection.

In addition to the aforementioned pathogenesis directly caused by DENV, mental health issues following infection could also be attributed to acute reactions triggered by unexpected life disruptions, particularly stress-associated symptoms such as anxiety, insomnia, and depression. Symptoms of dengue such as the severity of fever, headache, myalgias, arthralgias, and retro/periorbital pain were positively correlated with both anxiety and depression [15]. A pertinent example is the aftermath of coronavirus disease 2019 (COVID-19), survivors of this illness commonly reported fatigue, muscle weakness, sleep disturbances, and feelings of anxiety or depression. Intriguingly, these symptoms were especially pronounced in those who experienced severe illness during their hospital stay [38]. Hospitalization, inherently, can profoundly disrupt one’s daily routines. Amidst such upheavals, individuals confront exceptional stresses and might succumb to debilitating psychological issues like depression, anxiety, and insomnia [39]. Typically, negative life events steer individuals towards one of two trajectories: resilience, where the impact on anxiety or depression symptoms is negligible, or recovery, marked by an immediate surge in these symptoms but followed by a progressive improvement [40,41]. In the context of our research, we found that patients contracting dengue experience an elevated risk of depression. Notably, hospitalized dengue patients also had an increased short-term risk (<3 months) of anxiety and insomnia. Within Taiwan’s universal healthcare system, dengue patients receive comprehensive care designed to manage their condition throughout and after the infection, often at minimal cost. However, despite the high standard of care provided, these individuals may still encounter significant psychological stress, potentially leading to an elevated risk of developing mental health disorders subsequent to their infection.

Several key strengths of our study need to be addressed. First, ours is a population-based cohort 98study with a large sample size of laboratory-confirmed outpatient and inpatient dengue cases. Second, Taiwan has a universal healthcare system that covers more than 99% of the population. This comprehensive health system significantly minimizes loss to follow-up this cohort study. Third, this study accounted for many important potential confounders related to these three mental illnesses, including demographic and socioeconomic factors, as well as comorbidities. Finally, we accounted for multiple testing by applying the Benjamini-Hochberg procedure, minimizing the likelihood of false-positive findings.

There were several limitations in our study. Firstly, the diagnosis of these three mental disorders primarily relied on claims data from the NHI Program. To enhance the validity of the diagnosis, we chose only those patients who sought follow-up care in psychiatric clinics. Nevertheless, patients who did not seek medical help would not be detected in the NHIRD, potentially leading to an underestimation of the incidence. Secondly, some potential confounders linked to stress, such as alcohol consumption, education level, family history, violence, trauma, and other personal emotional events, were not available in the NHIRD. Thirdly, potential misclassification of nondengue subjects also poses a limitation, as DENV infections can be asymptomatic or result in mild symptoms, which may go unnoticed or underreported. However, this misclassification bias should not be large, given that dengue is currently not endemic in Taiwan, resulting in a low overall incidence and seroprevalence, with outbreaks usually confined to certain hot spots [20–22]. Fourthly, patients with secondary dengue infections generally face a higher risk of developing severe disease, which may influence the risk of subsequent mental health issues differently compared to those with primary infections. However, our analysis focused solely on primary dengue infections due to the limited number of cases identified with more than one laboratory-confirmed dengue infection in our database. Therefore, our study included new laboratory-confirmed dengue cases between 2002 and 2015 and excluded any dengue cases with a prior laboratory-confirmed diagnosis dating back to 1998. It is important to note that some of these cases might represent secondary DENV infections if serological data were available, presenting a limitation in our ability to accurately distinguish the effects between primary and secondary infections. Nevertheless, we presumed that the majority of included cases were primary infections, given that dengue is not endemic in Taiwan with a low overall incidence and seroprevalence [20–22]. Fifthly, until 2009, laboratory confirmation of dengue in our study involved the detection of DENV IgM or IgG in a single serum sample. This method could have led to some degree of misclassification due to cross-reactivity with other flaviviruses. Nevertheless, as dengue is not endemic in Taiwan and seroprevalence was low, especially before 2009, this source of bias is unlikely to impact our findings significantly.

Sixthly, we excluded 27,758 dengue patients, approximately 37.7% of our total cohort, due to prior diagnoses of mental health disorders, irrespective of the diagnosing physician’s specialty or the diagnosis setting. This comprehensive approach was designed to ensure our cohort was free from pre-existing mental illnesses, enabling an accurate assessment of new incidences. For defining outcomes, we implemented strict criteria requiring at least one hospital admission or three outpatient visits with relevant diagnostic codes, verified by board-certified psychiatrists. Although this stringent approach minimized misdiagnosis and misclassification, it might have also led to an underestimation of the risk of mental disorders post-DENV infection due to its conservative nature. Seventhly, our study includes participants younger than 18 years, with some as young as infancy. While these participants represent a small proportion of the study population and are unlikely to significantly affect our results, the inclusion of very young children—who are difficult to accurately diagnose for mental health disorders—may limit the precision of our findings regarding the incidence of these conditions. Finally, it is challenging to ascertain whether the increased hazards of depression are due to prior dengue infection, the experience of treatment or hospitalization, or a combination thereof. This is because our dengue cases were diagnosed and received treatment accordingly, while controls were selected from the general population, encompassing a broad spectrum of health statuses. Future large-scale cohort studies with different control groups with different health status or disease severity are required to elucidate whether the observed increased risks were specific to dengue, or could be a byproduct of any potentially serious illness.

In our study, we observed an increased risk for depressive disorders among patients diagnosed with dengue. This suggests a possible association between dengue infection and the subsequent development of depressive disorders. Future research is needed to explore the detailed mechanisms behind this association and to determine whether dengue directly contributes to the onset of depressive disorders.

## Supporting information

S1 Table List of ICD-9-CM and ICD-10-CM codes for identifying mental illness in this study. (DOCX)
