# Supplementary material for: Risks of anxiety disorders, depressive disorders, and sleep disorders in patients with dengue fever: A nationwide, population-based cohort study
Source: PLoS Negl Trop Dis. 2024 Jul 3;18(7):e0012239. doi: 10.1371/journal.pntd.0012239 (PMC11221675; doi:10.1371/journal.pntd.0012239)
Supplement: S1 Table — (DOCX) [file pntd.0012239.s001.docx]

**Supplementary Material Legend**

**S1 Table.** List of ICD-9-CM and ICD-10-CM codes for identifying mental illness in this study

**S1 Table.** List of ICD-9-CM and ICD-10-CM codes for identifying mental illness in this study

| **Mental illness** | **ICD-9-CM (until 2015)** | **ICD-10-CM (since 2016)** |
| --- | --- | --- |
| Sleep disorders | 780.50, 780.52, 780.54, 780.58, 780.59, 307.4 | F51, G47.0, G47.1, G47.2, G47.5, G47.6, G47.8, G47.9 |
| Sleep disturbance | 780.50 | G47.9, G47.2, G47.5 |
| Insomnia | 780.52 | G47.0 |
| Hypersomnia | 780.54 | G47.1 |
| Sleep-related movement disorder | 780.58 | F51, G47.6 |
| Other sleep disturbances | 780.59 | G47.8 |
| Anxiety disorders | 300 | F40, F41, F42, F44, F45, F48 |
| Phobic anxiety disorders |  | F40 |
| Other anxiety disorders |  | F41 |
| Obsessive-compulsive disorder |  | F42 |
| Dissociative and conversion disorders |  | F44 |
| Somatoform disorders |  | F45 |
| Other nonpsychotic mental disorders |  | F48 |
| Depressive disorders | 296.2, 296.3, 311 | F32, F33, F34.1 |
| Major depressive disorder single episode | 296.2 | F32 |
| Major depressive disorder recurrent episode | 296.3 | F33 |
| Depressive disorder, not elsewhere classified | 311 |  |
| Dysthymic disorder |  | F34.1 |

Abbreviation: ICD-9/10-CM = International Classification of Diseases, Ninth/Tenth Revision, Clinical Modification.

Specific code 780.50 Sleep disturbance, unspecified convert 780.50 to ICD-10-CM

Specific code 780.51 Insomnia with sleep apnea, unspecified convert 780.51 to ICD-10-CM

Specific code 780.52 Insomnia, unspecified convert 780.52 to ICD-10-CM

Specific code 780.53 Hypersomnia with sleep apnea, unspecified convert 780.53 to ICD-10-CM

Specific code 780.54 Hypersomnia, unspecified convert 780.54 to ICD-10-CM

Specific code 780.55 Disruption of 24 hour sleep wake cycle, unspecified convert 780.55 to ICD-10-CM

Specific code 780.56 Dysfunctions associated with sleep stages or arousal from sleep convert 780.56 to ICD-10-CM

Specific code 780.57 Unspecified sleep apnea convert 780.57 to ICD-10-CM

Specific code 780.58 Sleep related movement disorder, unspecified convert 780.58 to ICD-10-CM

Specific code 780.59 Other sleep disturbances
